# Strengths Model-Based Nursing Interventions for Inpatients in Psychiatric Inpatient Settings Using a Seclusion Room: A Case Series Study

**DOI:** 10.3390/nursrep13020057

**Published:** 2023-04-09

**Authors:** Yutaka Nagayama, Koji Tanaka, Masato Oe

**Affiliations:** 1 School of Nursing, Kanazawa Medical University, 1-1 Uchinada, Kahoku 920-0265, Japan; 2 Faculty of Health Sciences, Institute of Medical, Pharmaceutical and Health Sciences, Kanazawa University, Kanazawa 920-0942, Japan

**Keywords:** strengths model, minimizing coercive measures, seclusion, collaborative relationship, recovery-oriented care

## Abstract

The use of coercive measures in psychiatric inpatient settings has been an important issue for many years. Nursing interventions based on a strengths model could enable a reduction in the use of these measures. This study aimed to describe the practice of nursing interventions using a strengths model for psychiatric inpatients who have been in seclusion for a long time. We also constructed a nursing model to minimize coercive measures. The participants were eight inpatients who had been in seclusion for a long time. Nursing interventions based on a strengths model were implemented in collaboration with nurses from six long-term care units in three psychiatric hospitals in Japan. For 4 of the 8 participants, the seclusion time decreased by 20–45%. However, for another 2, it increased by about 23–34%. An average decrease of 9.6% was observed, and the open observation time increased by 1.4 h per day on the seclusion days. When using this model, the nurses considered the effects of stimulating strengths. We believe this approach may promote inpatients’ self-insight. Considering the perspective of stimulus adjustment might be useful for maximizing the positive effects of working on strengths.

## 1. Introduction

Prolonged seclusion and physical restraint have become a serious problem in Japanese inpatient psychiatric care. In a survey of inpatients who had undergone seclusion or physical restraint in six acute wards of four psychiatric hospitals in Japan, the median seclusion time was 204 h, and the median physical restraint time was 82 h [1]. In Japan, the periods of seclusion and physical restraint tend to be prolonged compared with other countries [2]. Regarding the relationship between coercive measures and nurse–bed ratios in Japanese psychiatric inpatient settings, as the number of nurses per 10 beds increased, the odds ratios for seclusion and physical restraint increased from 1.74 to 2.36, and an increase in the nurse–bed ratio did not lead to the avoidance of seclusion or physical restraint [3]. Instead, inpatients who need seclusion and physical restraint are typically hospitalized in wards with a high nurse–bed ratio, such as psychiatric emergency wards, and it is predicted that most inpatients will undergo seclusion and physical restraint.

In Japan, inpatients with severe mental disorders tend to be hospitalized for a long period. Some are kept in seclusion rooms in long-term care units because of persistent agitation, violence, and causing nuisance to other inpatients. To end the use of coercive measures, nurses in Japan’s long-term care units have performed “care aimed at avoiding mental and physical exhaustion”, “standardized care that does not confer a disadvantage to patients”, and “immediately responding to prevent problematic behaviors” [4]. However, challenging behavior related to the long-term use of seclusion rooms continues and many care environments hinder inpatients’ sociability and autonomy.

Various interventions have been developed to reduce the use of seclusion and restraint, which run the risk of causing negative effects for both inpatients and medical staff. The effects of these interventions have been verified [5]. For example, a trauma-informed care (TIC) approach reduced seclusion and restraint by 67% by establishing a therapeutic environment [6]. In addition, the “Six Core Strategies to Reduce the Use of Seclusion and Restraint” advocated by the National Association of State Mental Health Program Directors was constructed on the basis of patient-centered and strengths-based care using TIC [7]. These six core strategies have been reported to reduce physical restraint and seclusion by 62–86%, enable two-way communication with psychiatric inpatients, and improve decision-making for psychiatric inpatients [8]. In the safe wards model, nurses choose to reduce and prevent conflict-causing factors and not use coercive measures [9]. A pragmatic cluster-randomized controlled trial using the safe wards model was conducted in psychiatric inpatient settings and found a 15% reduction in conflict situations and a 26.4% reduction in the use of coercive measures [10]. In another study, mindfulness-based stress reduction training was conducted with nurses for 2 months. Safety between nurses and inpatients improved, the need for one-on-one contact decreased, and the use of physical restraints decreased [11].

Strengths-based care is already incorporated as an element of various interventions that avoid seclusion and physical restraint. It has been reported to improve mood (including depression, anger, and retention-anxiety) and contribute to cultural changes in the therapeutic environment beyond diagnosis and medication [12]. In the field of mental health welfare, the use of a strengths model that draws out the strengths of chronic mental illness inpatients and promotes recovery is emphasized [13]. In strengths-based case management, supporters accompany the client, respect the client’s decision-making, and support the individual to achieve their hopes and goals [14]. We speculated that the use of the strengths model might facilitate problem-solving regarding the long-term use of seclusion rooms for inpatients with severe mental disorders. By transforming the relationship to utilize the strengths of patients with severe mental disorders who have been placed in seclusion rooms for a long period of time, the autonomy and recovery of the inpatient could potentially be promoted, and coercive measures could be reduced.

The purpose of this study was to describe the practice of nursing interventions using a strengths model for psychiatric inpatients who had been placed in a seclusion room for a long time. The research questions in this study were: how do nursing interventions using a strengths-based model affect the use of coercive measures for psychiatric inpatients who have been using a seclusion room for a long time, and how can these interventions be implemented for them? Furthermore, on the basis of the responses of individual cases to nursing interventions, we constructed a nursing model for minimizing coercive measures that utilizes the strengths of inpatients in the seclusion room.

## 2. Materials and Methods

### 2.1. Research Design

Case series study.

### 2.2. Participants

The participants were eight inpatients who had been placed in a seclusion room over a long period. The selection criteria were used to recruit inpatients who continuously required seclusion or physical restraint in a seclusion room for more than 1 month or who had intermittently repeated implementation and release in the past 3 months. We excluded inpatients who were subjected to physical restraint and seclusion for the treatment of physical complications and those who had difficulty understanding or required safety management for restless states caused by delirium and dementia.

All the participants had previously received an assessment of their behavioral problems and had been provided with a nursing intervention focused on controlling their behavioral problems.

### 2.3. Education for Nurses about the Strengths-Based Model

From April to July 2017, we conducted teaching on the strengths-based care model for nurses at the research facilities. We used the strengths model of Rapp and Goscha [13] and explained the purpose and main concepts of the model to the nurses. The nurses were trained in assessment using the strengths model, considering cases of inpatients in a psychiatric inpatient setting. The authors and nurses jointly extracted the inpatients’ strengths and worked using a “strengths assessment sheet” to examine the specific content of the nursing intervention. As a formal part of the assessment, we examined the strengths of the inpatient and the strengths of the environment surrounding them across three aspects: current strengths; future wishes, desires and aspirations; and past resources. The strength domains had seven elements of personal and environmental strength (daily life, economic aspects, work/education/specialized knowledge and skills, relationship with supporters, comfort/health, leisure, and culture/values/beliefs).

Next, we explained how to use the patients’ strengths that had been identified during the assessment in nursing interventions. As a concrete intervention procedure, the nurses share strengths through dialogue with inpatients and repeat discussions with the inpatients about how they can perform strengths-based activities.

Focus group interviews on the strengths emphasized nurses who had experience working with patients to create perspectives on diverse patient strengths. The patients were not included in the focus group interviews because there was concern that multiple nurses discussing various strengths would confuse the patients and increase their mental stress. However, the strengths that were discussed in the group interviews with multiple nurses were shared by the nurses through dialogue with the patients after the group interviews, and the nurses discussed with the patients how to use the strengths.

### 2.4. Implementation of Strengths Model-Based Nursing Interventions

From August 2017 to March 2018, we conducted monthly focus group interviews with the nurses in conference rooms at the inpatient settings. The total number of group interviews was 5–7. In the first group interview, we discussed the identified strengths of the target patients and developed interventions that made use of their strengths. After the group interview, the nurses had conversations with the inpatients about their strengths and how to use them.

In the second and subsequent group interviews, the authors and nurses reflected on the nursing intervention for the inpatient, assessed the strengths of the individual inpatient and their environment, evaluated the inpatient’s response to nursing interventions using the strengths model, and confirmed the direction of specific nursing interventions for inpatients. After each group interview, the nurses repeatedly shared strengths with the patients through dialogue, helping the patients to use their strengths autonomously. The group interview time was approximately 30 min per inpatient. The completion of the nursing intervention using the strengths model was judged comprehensively on the basis of the effects and issues of the nursing intervention. The intervention term was 171–238 days.

### 2.5. Data Collection

Inpatient demographics information was collected from medical records including gender, age, diagnosis, duration of mental illness, and number/length of hospitalizations. Data regarding coercive measures were collected as the number of instances of seclusion, the total number of days of seclusion, and the reasons for seclusion in the past year. We also collected data on the number of days without seclusion, total seclusion time, open observation time during seclusion each day, and antipsychotic drug dosage for participants before and after the intervention period. Additionally, the monthly group interviews with the nurses were recorded using a voice recorder and transcribed verbatim.

In the focus group interviews, we investigated how patients’ strengths were determined, how the nursing intervention was planned, how the patients’ response to the strengths-based nursing intervention was evaluated, and how the strengths-based nursing intervention affected the use of coercive measures.

### 2.6. Data Analysis

We compared the number of days without seclusion, total seclusion time, and open time during seclusion each day before and after the intervention period for each individual case.

From verbatim transcripts of the group interviews with the nurses, we described how the nurse perceived the strengths (daily life, economic aspects, work/education/specialized knowledge and skills, relationship with supporters, comfort/health, leisure, and culture/values/beliefs) in each case. Next, we described how the nurse implemented nursing interventions aimed at minimizing coercive measures and how the inpatient responded.

The core meaning of the nursing intervention designed to minimize coercive measures using the strengths model for inpatients who were placed in seclusion rooms for a long time was identified from “nursing judgment based on the strengths of the inpatient” and “nursing intervention based on strengths,” and categories were generated. A nursing model for minimizing coercive measures utilizing the inpatients’ strengths in the seclusion room was created by examining the relationships between multiple categories.

### 2.7. Ethical Considerations

This study was conducted with approval (I133) from the Medical Research Ethics Review Committee of Kanazawa Medical University. We explained the research plan verbally and in writing to the inpatients who were invited to participate and their families and obtained their consent. Participation in this research was voluntary, and the patients did not suffer any disadvantage for declining to participate. If deterioration of the participants’ mental condition occurred during the strengths model-based nursing intervention, we planned to suspend or discontinue the study.

## 3. Results

### 3.1. Participants’ Demographic Information (Table 1)

The inpatients were four men and six women. Their mean age was 48.6 ± 10.7 years, and the mean duration of their mental illness was 28.1 ± 13.0 years. The most common diagnosis was schizophrenia, which was seen in six of the eight patients. The mean number of hospitalizations was 5.4 ± 5.0, and the mean length of stay was 13.7 ± 14.0 years. The mean number of seclusions in the past year was 3.0 ± 2.1, and the mean seclusion period (total number of days) in the past year was 160.3 ± 119.3 days. 

The chlorpromazine equivalency values (mg) for antipsychotic medication before and after the strengths-model-based nursing interventions are shown in Table 1. The mean chlorpromazine equivalency values were similar at 537 mg pre intervention and 555 mg post the strengths-model-based intervention. Four patients (B, C, D, and F) were treated with a single second-generation antipsychotic before the nursing interventions. Two patients (A and E) were treated with a double second-generation antipsychotic before the nursing interventions. Five patients (A, B, F, G, and H) were treated with first-generation antipsychotics (e.g., zotepine, levomepromazine, and chlorpromazine) to suppress sedation for psychomotor agitation. Five patients (B, C, E, G, and H) were treated with a mood stabilizer (sodium carbonate and lithium carbonate). After the strengths-model-based nursing interventions, two patients (B and D) changed from a single second-generation antipsychotic to a double drug combination. One patient (E) changed from a double second-generation antipsychotic to a single drug.

**Table 1:** Participants’ demographic information.

Case	Gender	Diagnosis	Length of Hospitalization at the Start of the Study	Number of Instances of Seclusion in the Past Year	Total Number of Days of Seclusion in the Past Year	Reason for Seclusion	Chlorpromazine Equivalency Values (mg) before Intervention	Chlorpromazine Equivalency Values (mg) after Intervention
A	Male	Schizophrenia	16 years and 3 months	1	206	Agitation, restlessness	1257	1257
B	Male	Schizophrenia	11 years and 2 months	1	177	Violence towards other patients, agitation, restlessness, self-harm	905	1205
C	Female	Schizophrenia	36 years	6	145	Agitation, restlessness, violence towards others, nuisance to other patients	159	23
D	Female	Intellectual disability	4 years and 4 months	4	81	Agitation, restlessness, nuisance to other patients	0	41
E	Female	Schizophrenia	32 years and 7 months	1	350	Agitation, restlessness, water intoxication	988	988
F	Male	Schizophrenia, intellectual disability	31 years and 9 months	5	119	Agitation, restlessness, nuisance to others	400	215
G	Female	Autism spectrum disorder, intellectual disability	2 years and 3 months	6	112	Self-harm, suicide attempt	587	400
H	Female	Schizophrenia, anorexia nervosa	3 years and 9 months	1	365	Violence towards others, nuisance to others	94	185

### 3.2. Changes in Seclusion Days, Seclusion Time, and Open Observation Time before and after the Nursing Intervention Based on the Strengths Model (Table 2, Table 3 and Table 4)

After the introduction of the strengths model, the number of days without seclusion increased in five cases (cases A, B, C, E, and H). 

**Table 2:** Number of days without seclusion before and after nursing intervention based on the strengths model.

Case	Intervention Period (Days)	Before Intervention	After Intervention
A	220	0	22
B	177	0	103
C	232	97	152
D	238	157	0
E	196	0	55
F	171	41	25
G	171	58	3
H	171	0	10

**Table 3:** Comparison of total seclusion time (hours) before and after nursing intervention based on the strengths model.

Case	Before Intervention		After Intervention	
	Total Seclusion Time	Ratio	Total Seclusion Time	Ratio
A	3634	68.8	3082	58.4
B	3488	82.1	1577	37.1
C	2149	58.5	886	24.1
D	1585	27.7	3502	61.3
E	3654	77.7	2364	50.3
F	2311	56.3	2484	60.5
G	2514	61.3	3455	84.2
H	3297	80.3	2473	60.3

**Table 4:** The average open observation time (hours) per day before and after nursing intervention based on the strengths model.

Case	Before Intervention	After Intervention
A	7.5	8.4
B	4.3	2.7
C	5.1	6.1
D	4.4	9.3
E	5.4	7.3
F	8.2	7.0
G	1.6	3.4
H	4.7	8.6

The ratio of total seclusion time in all cases was 64.1% on average before the nursing intervention based on the strengths model, and the total seclusion time after the nursing intervention decreased by 54.5%. The cases in which there was a decreased seclusion time were case B (45% decrease), case C (34.4% decrease), case E (27.4% decrease), case H (20.0% decrease), and case A (10.4% decrease). Conversely, the total seclusion time increased in case D (33.6% increase), case G (22.9% increase), and case F (4.2% increase).

The average open observation time per day was 5.2 h before the nursing intervention, and for the time after the nursing intervention, it increased to 6.6 h.

### 3.3. Nursing Model for Minimizing Coercive Measures Utilizing Strengths of Inpatients in the Seclusion Room (Figure 1)

Table 5 shows the strengths of the inpatients, the nursing intervention based on the strengths, the inpatients’ response, and the influence on seclusion use for each participant. We then built a nursing model designed to minimize coercive measures and use inpatients’ strengths in the seclusion room, as follows.

**Figure 1:**
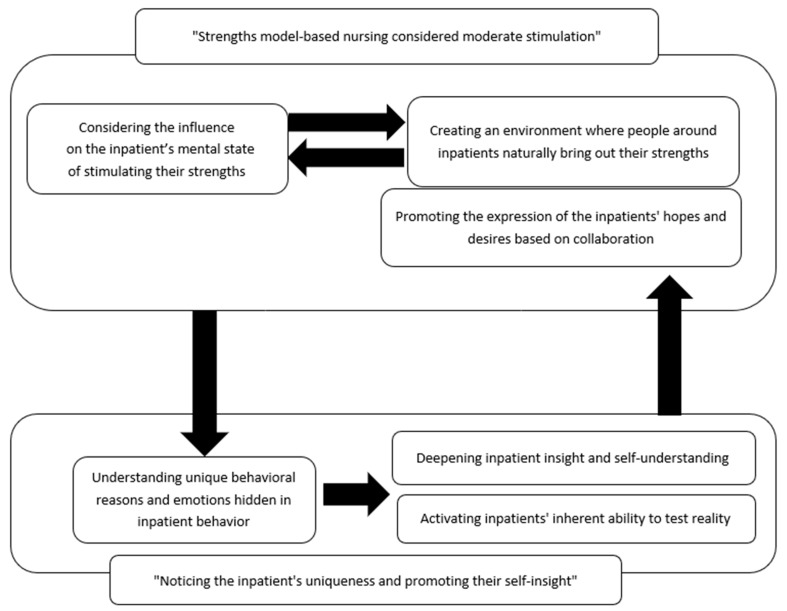
Nursing model of minimizing coercive measures utilizing strength of inpatient in seclusion room.

In the category “Utilization of strengths considered moderate stimulation”, after determining whether the mental state of the inpatient was disturbed by stimulation resulting from the nurses’ intervention based on the strengths model, the nurses encouraged the expression of the inpatients’ hopes and desires and created an environment in which people around the inpatients naturally drew out their strengths. In the category “Noticing the inpatient’s uniqueness and promoting the inpatient’s self-insight”, the nurses considered the unique reasons and emotions underlying the inpatients’ intense and destructive behavior. The inpatients therefore gained deeper insight into their own intense emotional changes and engaged in a relationship that activated their inherent reality-testing ability. The two categories interacted with each other, and the nurse tried to build a recuperation environment that minimized coercive measures for inpatients (Figure 1). The sub-concepts of these two categories are described below.

**Table 5:** Strengths of inpatients, nursing intervention, inpatient responses and influence on seclusion.

Case	Strengths of Inpatients	Nursing Interventions Based on Strengths	Inpatient Response and Influence on Seclusion
A	Goodwill and a desire to help others, recognized the emotions of others and experienced them as if they were his own (relationship with supporters). Knew his boombox was something valuable and important to him (culture/values/beliefs). Realized that his behavior was not socially acceptable even when he was confused (culture/values/beliefs).	When sensing that the patient was giving signs to others, trying to understand his feelings and calling out to him so that he could express his feelings as much as possible. When the patient was in a state of confusion, giving a frank and realistic call to action.	Told the nurse that his destructive behavior was because he superimposed his feelings on the music and expressed emotions that he could not contain. There was a phase when he calmed down and his confused behavior subsided. He was able to stay out of seclusion during this phase.
B	Music-related activities. Exaggeratedly said that he used to be a singer and made a lot of money (leisure). Sometimes expressed his wishes by saying things like “I want to eat cutlet curry” (leisure). Used to play softball on the grounds of the facility to exercise (leisure).	Identifying the right time to talk about music by observing the patient’s mental state. Suggesting that the patient’s goal was to go out and eat delicious food. Thinking that participation in the exercise program would lead to a sense of camaraderie and improve the patient’s ability to test reality, and therefore recommending participation in futsal as a ward activity.	Gradually became more active and could go and stay with his family overnight. Didn’t seem to escalate his desire for things after staying out. The nurse discussed future goals with him, and he decided to go to his house for a sleepover on New Year’s Day. He felt pressure to go to sleepovers, and was placed in seclusion again. A few days after visiting a futsal club, became talkative, and expressed the delusion that he was in an intimate relationship with another patient.
C	Checking and consulting with nurses (relationship with supporters). In the past, was sociable, clinging to others and having the energy to actively interact with them (relationship with supporters). Putting up with her current diet and restrictions on snacks (comfort/health). Writing her weekly schedule in her notebook (daily life). She was meticulous and liked to write (culture/values/beliefs).	Observation was focused on what the patient said about her desires about her diet. Encouraging the patient’s activities, such as accompanying her to gymnastics, and providing meals that were similar to those of others as a reward for being able to do activities. Providing the patient with snacks that were less likely to worsen her abdominal condition and that would make her feel more full. Bringing leaflets, notebooks, and ballpoint pens into her room so she could write whatever she wanted.	Talked with other inpatients and expressed herself calmly. At times, her mental state became unstable, and she exhibited behavior such as begging other patients for sweets or going into the nurse’s center and looking for snacks to eat. Seclusion was required only when there was aggressive behavior and she ate other inpatients’ food even though her abdominal symptoms worsened. When her snack pattern broke, was on the verge of becoming violent. However, her agitation subsided as her nurse tried to arrange snacks. Drew a character and looked at leaflets and newspapers.
D	Was aware that she was tearing up the newspaper, and it felt like a plaything. Would neatly disassemble and tear up anything she put in her room (leisure). In the past, had been interested in clothes and hairstyles, and she used to style her own hair. Now, when going out of the ward, was concerned about people seeing her, and would wear clothes over her shirt. After taking a bath, seemed to carefully choose from among her few clothes (daily life). Intended to clean up her room (daily life).	Creating a toolbox to put away newspapers in collaboration with the patient. Responding to the patient’s requests as much as they could, and bringing her magazines. In line with the patient’s requests, bringing a TV, a chest of drawers, and other items to her room, to incorporate more of the experience of the patient making her own choices into her daily life.	Felt that if she told her nurse that there was a problem, the nurse would do something about it. In the process of making the toolbox, actively participated in the process of selecting origami, applying glue, and selecting tape. When she continued to receive magazines from nurses, was unstoppable when her demands escalated. After starting the strengths model intervention, required seclusion for most of the day. Was particular about the layout of her room. Behavior of destroying things continued, such as cutting the wires of the TV and disassembling it into small pieces.
E	Her mother was in a different ward of the same hospital. She doesn’t talk much these days, but they used to see each other from time to time (relationship with supporters). Was friendly towards other patients (relationship with supporters). Stopped showing strong resistance to nurses (relationship with supporters). Liked to sing, and always sang at karaoke in the ward (leisure).	Proposed that the patient should visit her mother’s ward and go to the shop accompanied by a nurse. Trying to make time for the patient to spend time outside her ward so she could change her mind. Trying to speak softly and soothingly about her behavior of drinking excessive amounts of water. Being careful not to engage in any restraining behavior in the relationship.	Began participating in occupational therapy and expressed a desire to return home. When she drank a lot of water, nurses told her softly, “Be careful not to drink too much.” When nurses approached her with a gentle demeanor and jokes, she softened her expression. She no longer showed anger or violence toward the nurses. The open observation time was gradually extended, and most of her day was spent without seclusion. When she felt negative feelings or when she was eating, would go into her room.
F	Didn’t lie and was honest about what happened. Always said “I’m sorry” after showing any problematic behavior (relationship with supporters). Appealed to nurses to take care of him (relationship with supporters). Liked playing cards, Othello, reading newspapers, and watching high school baseball and singing programs on TV (leisure). Communication was problematic in terms of social norms, but would try to get close to the other person and make them happy (culture/values/beliefs).	Assuming that he truly wanted to get along with others and was taking action by choosing this means of communication, trying to consider releasing him from seclusion. Continuing mastication training in cooperation with speech pathologists. Making time for the patient’s favorite hobby and encouraging him to participate in occupational therapy.	Medical staff considered the option of behavioral observation in a general room, and seclusion was temporarily lifted. Knew that female patients and staff would get angry, but still touched them. Seemed to recognize that going into other patients’ rooms was a bad idea, and seemed to be able to control his compulsions a little. Performed swallowing exercises every day, but didn’t really see the need. Didn’t have much interest in occupational therapy, so didn’t say that he wanted to participate.
G	Felt that the nurses didn’t refute her story at all and accepted her. When unable to express her feelings to her doctor, often told the nurse later (relationship with supporters). Liked to write and give her nurses notes describing her thoughts. Was comfortable when painting (comfort/health). Liked to draw, and in the past had made postcards and sold them (leisure). Wanted to be physically instead of mentally disabled (culture/values/beliefs).	Making time to convey their thoughts, even if time was short, such as spending 5 min sharing a story about the day. Encouraging her to spend more time on creative activities, which she is good at. Developing a shared understanding when there was a gap between the patient’s and nurse’s perceptions of nuances. Clarifying what they could and could not do. Listening to what she said about the kind of life she wanted to live, and how she wanted to spend her open observation time.	Was not good at taking action on her own, but sometimes accepted the suggestions of her nurse. Self-harm continued and open observation time was temporarily shortened when self-injury occurred. However, was sometimes able to spend time without self-harming. Developed a method for sending out an SOS when she was in distress. Learned to wait without exploding when anxious. In the notebook in which she wrote down her thoughts, stopped expressing her frustration even when there was no immediate response from her head nurse.
H	Participated in the SST role-play, and looked happy when praised by other inpatients or received feedback (relationship with supporters). Gathered with other female inpatients in the hall, and talked about fashion and music (relationship with supporters). Wanted to live alone, or to go shopping with her mother and grandmother (comfort/health). Even though she experienced somatic hallucinations, was not completely incomprehensible (comfort/health). Understood treatment policy on weight and diet (comfort/health). Liked to read fashion magazines and admired models with her ideal body type (culture/values/beliefs).	Encouraging the patient to verbalize her hopes for the future. Trying to build a relationship that gave her hope about the possibility of going shopping together when the seclusion was lifted. When she felt sensory hallucinations, making clear that it wasn’t actually happening. Giving a specific amount of water, which was written on a checklist. Trying their best to understand her requests. Providing feedback about what she was able to do and what she could do in the past even though she could not do it now.	When her feelings were heightened because her desires were not fulfilled, took measures such as leaving the area, or going to her room and shouting loudly. By consulting with nurses, became less obsessed with eating or not eating. Became able to express her own feelings honestly, and was able to spend more time outside seclusion. After using abusive language to a male nurse, said, “I misrepresent my feelings by verbally abusing a male nurse. I feel bad for the male nurse.” Often complained that she wanted to live alone and go shopping after discharge from hospital. Began asking other patients what they would do after discharge.

### 3.4. Subcategories of “Strengths-Model-Based Nursing Considered Moderate Stimulation”

#### 3.4.1. Considering the Influence on the Inpatients’ Mental State of Stimulating Their Strengths

The nurses focused on enabling the inpatients to have an enjoyable time through nursing interventions that made use of their strengths. However, they feared that inpatients’ demands would escalate and develop into challenging behavior. For this reason, the nurses determined the degree of disturbance in the inpatient’s mental state caused by stimulating their strengths.

#### 3.4.2. Creating an Environment in Which People around Inpatients Naturally Bring Out Their Strengths 

Other inpatients and nurses around the inpatient naturally communicated with each other to make the inpatient feel comfortable. A therapeutic atmosphere was created in which the recuperation environment of the inpatient itself brought out the strengths of the inpatient.

#### 3.4.3. Promoting the Expression of the Inpatients’ Hopes and Desires through Collaboration

Through collaborative activities with the inpatients, the nurses were able to elicit discussion of hopes and desires that the inpatients had not expressed before. The nurses built relationships that allowed the inpatients to actively express their intentions. Individual collaboration informed upon what the inpatients wanted to do next, and they gradually expanded their perspective about their lives.

### 3.5. Subcategories of “Noticing the Inpatient’s Uniqueness and Promoting the Inpatient’s Self-Insight”

#### 3.5.1. Understanding the Unique Behavioral Causes and Emotions Underlying Inpatient Behavior

When an inpatient develops a challenging behavior that can be an indication for seclusion, the nurses gain deeper insight into what the inpatient places value on, based on the inpatient’s understanding of their own behavior. The nurses try to make sense of the inpatient’s behavior by focusing on the inpatient’s own description of the reasons behind their behavior.

#### 3.5.2. Deepening Inpatient Insight and Self-Understanding

The nurses gave positive feedback that the inpatient was able to implement and worked to increase the inpatients’ sense of self-affirmation. The inpatients then expressed their own emotional experiences to the nurse, and their self-understanding deepened.

#### 3.5.3. Activating the Inpatients’ Inherent Ability to Test Reality

The inpatients tended to have difficulties in two-way communication, such as difficulty in conveying their intentions to the others and perceiving others’ stories in a damaging manner, as well as biased interpretation. Therefore, the nurses were involved in helping the inpatient self-reflect on their own behavior and think about socially acceptable behavior.

## 4. Discussion

For patients with severe mental disorders who were placed in a seclusion room for a long period, nursing interventions based on a strengths model were implemented in collaboration with nurses from six long-term care units in three psychiatric hospitals in Japan. For 4 of the 8 participants, the seclusion time decreased by 20–45% during the study period. However, it increased for two participants by about 23–34%. An average decrease of 10.4–45.0% in seclusion time was observed in cases where coercive measures decreased after the study intervention. Changes in the antipsychotic dosage given to the participants were similar before and after the nursing interventions. However, all of the participants received first-generation antipsychotics or mood stabilizers for sedation during all of the periods. After the intervention, seven of the eight required dual second-generation antipsychotic combinations. This drug administration situation indicates that the patients’ sensitivity to external stimuli and vulnerability to exacerbation of psychiatric symptoms persisted, even after the intervention. The inpatients’ mental condition was unstable, and their desires and aggressive behaviors were sometimes escalated by nursing interventions that used their strengths. Therefore, considering the effects of stimulus adjustment is important for providing a unique perspective for maximizing the positive effects of working on strengths in the care of inpatients who have been placed in a seclusion room for a long period of time. The cases examined in this study were characterized by repeated aggressive behaviors involving positive symptoms, such as delusions of persecution, impulsive emotional outbursts, and sudden verbal abuse/violence. In addition, some inpatients expressed their desires, but had recently become less clear in expressing their own intentions and feelings. We also observed some cases in which the patient’s feeling of self-pity regarding their situation was increased, and their self-esteem was reduced. Positive and negative symptoms of schizophrenia inpatients have been shown to be associated with disability length, severity, self-stigma, and reduced self-directedness and cooperativeness [15]. In addition, in cases of cognitive dysfunction, it is characteristically difficult for patients to pay attention appropriately to continue conversations and build intimate relationships when interacting with others [16,17].

Compared with intervention studies such as those involving the TIC and Six Core Strategies approaches [5,7], the rate of reduction of coercive measures in the current study was small. All cases in this study were inpatients with schizophrenia whose conditions were severe and chronic and involved prolonged hospitalization. By continuing interventions based on the strengths model in addition to conventional care, it is possible that changes in the relationship with the nurse and activity content led to fluctuations in mental status and that coercive measures repeatedly increased and decreased during the intervention period. In addition, in the three cases in which the total seclusion time increased, it was found that the patients could not control their desires and continued to exhibit socially problematic behavior and sought to perform activities in their own interest without restrictions. Regarding background characteristics, all three of these cases had an intellectual disability, and it is possible that these disability characteristics increased the difficulty of releasing the inpatient from seclusion. Hyperactivity and stereotyped behavior, impulsiveness and restricted preferences, and impulsive and repetitive speech are characteristics associated with people with an intellectual disability [18].

Although the participants in this study exhibited mental instability and vulnerability, nursing interventions based on the strengths model showed a tendency to decrease the seclusion period overall. These results suggest that it may be useful for nurses to implement strengths-based care from a long-term perspective, without being bound by short-term fluctuations in the inpatient’s mental state caused by strengths-based care. Strengths-based care has been reported to contribute to the recovery of cognitive function and to complement conventional problem-solving approaches [19]. Strengths-based care can also develop psychiatric patients’ strengths, talents, and abilities, support patients to discover new activities they can engage in, and strengthen their self-esteem [20]. It has been reported that the long-term use of strengths-based care can reduce psychiatric patients’ self-stigma and promote personal recovery [21,22].

As shown in the nursing model for minimizing coercive measures by utilizing the strengths of the inpatient in the seclusion room constructed in the current study, developing cooperative relationships through strengths-based care allows inpatients to feel secure in the presence of nurses and makes it easier for nurses to understand inpatients’ emotions. Communication based on the strengths model focuses not only on strengths, but also on aspects of the patients’ vulnerability and distress [23]. The inpatient can then collaborate with surrounding supporters to build partnerships to think together about how the inpatient can deal with difficulties and problems [24]. Building collaborative relationships based on the strengths model may avoid factors that evoke challenging behavior in patients and help subjects to live autonomously and cooperatively.

## 5. Limitations

The data were limited to eight cases. The results of the current study represent an exploratory analysis of factors that may affect the reduction of coercive measures using the strengths model, and there may be limits to the generalizability of the findings. In the future, it will be necessary to set up a control group and verify the effectiveness of the strengths model for reducing coercive measures.

In the structure of the intervention of this study, after examining the strengths of the target inpatient in a group interview with the researcher and the nurse, the nurse conducted an intervention based on the inpatient’s strengths. Following the principle of the recovery strengths model, it is important to work together with the inpatient from the stage of examining their strengths. Therefore, it should be noted that this research is the result of modifying the framework of the strengths model. It is also possible that when the researchers pointed out the prolonged use of seclusion, this may have influenced the nurses to become more aware of coercive measures and consciously work to reduce seclusion.

In addition, it will be necessary for future studies to verify the effect on the reduction of coercive measures by nursing interventions on the basis of collaborative work with inpatients at all stages of intervention, in accordance with the strengths model.

## 6. Conclusions

For 4 of the 8 participants, the seclusion time decreased by 20–45%. However, it increased for 2 participants by about 23–34%. An average decrease of 9.6% in the seclusion time was observed, and the open observation time increased by an average of 1.4 h per day on seclusion days. It is therefore possible that a nursing intervention using a strengths model for inpatients who had been in seclusion for a long time might affect the term of seclusion.

We developed a nursing model for minimizing coercive measures utilizing the strengths of inpatients in the seclusion room. Using this model, nurses consider the effects of stimulating inpatients’ strengths and may promote inpatients’ self-insight. Considering the perspective of stimulus adjustment might be useful for maximizing the positive effects of working on strengths.
